# IgM-associated gut bacteria in obesity and type 2 diabetes in C57BL/6 mice and humans

**DOI:** 10.1007/s00125-022-05711-8

**Published:** 2022-05-19

**Authors:** James A. Pearson, Heyuan Ding, Changyun Hu, Jian Peng, Brittany Galuppo, F. Susan Wong, Sonia Caprio, Nicola Santoro, Li Wen

**Affiliations:** 1 Section of Endocrinology, School of Medicine, Yale University, New Haven, CT USA; 2 Division of Infection and Immunity, School of Medicine, Cardiff University, Cardiff, UK; 3 Department of Endocrinology, Shanghai Fifth People’s Hospital, Fudan University, Shanghai, China; 4 Adept Therapeutics, Inc., Beverly, MA USA; 5 Department of Pediatrics, School of Medicine, Yale University, New Haven, CT USA

**Keywords:** B cell, Gut bacteria, IgM, Obesity, Type 2 diabetes

## Abstract

**Aims/hypothesis:**

IgM is the primary antibody produced by B cells and we hypothesise that IgM antibodies to gut microbiota may play a role in immunometabolism in obesity and type 2 diabetes. To test our hypothesis, we used B6 mice deficient in activation-induced cytidine deaminase (*Aid*^−/−^ [also known as *Aicda*^−/−^]) which secrete only IgM antibodies, and human faecal samples.

**Methods:**

We studied the immunometabolic effects and gut microbial changes in high-fat-diet-induced obesity (HFDIO) in *Aid*^−/−^ B6 mice compared with wild-type mice. To determine similarities between mice and humans, human stool samples were collected from children and adolescents who were obese with normal glucose tolerance (NGT), obese with glucose intolerance (IGT), or obese and newly diagnosed with type 2 diabetes, for faecal microbiota transplant (FMT) into germ-free (GF) B6 mice and we assessed IgM-bound bacteria and immune responses.

**Results:**

Compared with wild-type mice, *Aid*^−/−^ B6 mice developed exacerbated HFDIO due to abundant levels of IgM. FMT from *Aid*^−/−^ B6 to GF B6 mice promoted greater weight gain in recipient mice compared with FMT using wild-type mouse faecal microbiota. Obese youth with type 2 diabetes had more IgM-bound gut bacteria. Using the stools from the obese youth with type 2 diabetes for FMT to GF B6 mice, we observed that the gut microbiota promoted body weight gain and impaired glucose tolerance in the recipient GF B6 mice. Importantly, some clinical features of these obese young individuals were mirrored in the GF B6 mice following FMT.

**Conclusions/interpretation:**

Our results suggest that IgM-bound gut microbiota may play an important role in the immuno-pathogenesis of obesity and type 2 diabetes, and provide a novel link between IgM in obesity and type 2 diabetes in both mice and humans.

**Data availability:**

The 16s rRNA sequencing datasets supporting the current study have been deposited in the NCBI SRA public repository (https://www.ncbi.nlm.nih.gov/sra; accession no. SAMN18796639).

**Graphical abstract:**

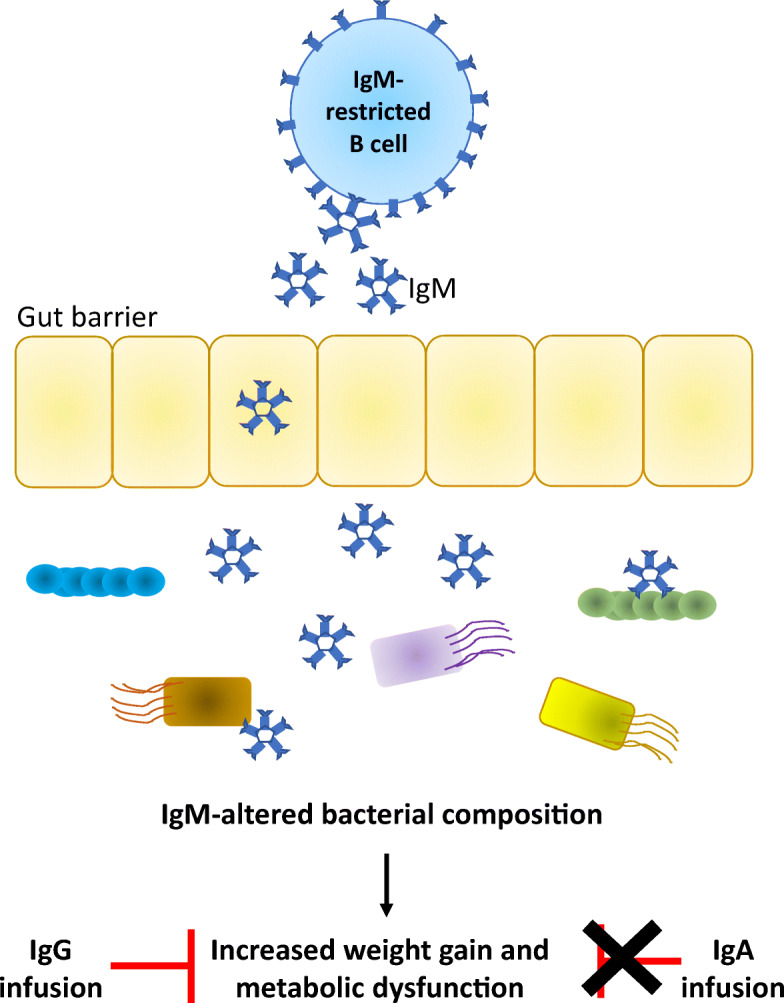

**Supplementary Information:**

The online version contains peer-reviewed but unedited supplementary material available at 10.1007/s00125-022-05711-8.

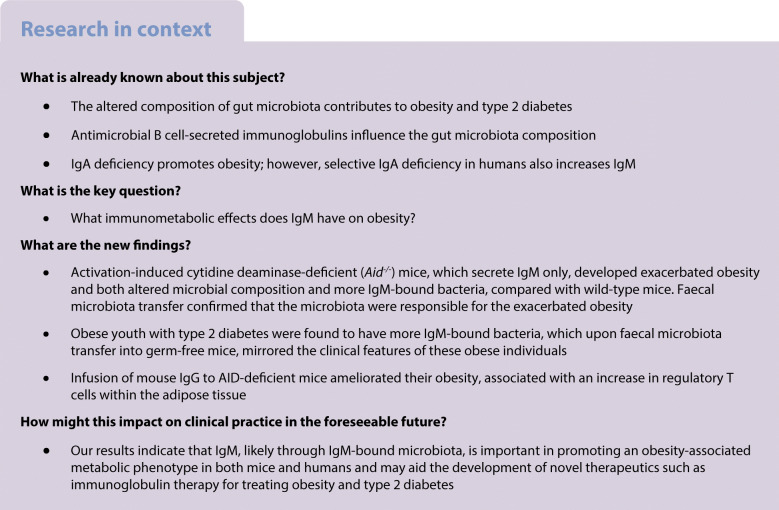



## Introduction

Obesity is a serious health problem worldwide. The US Centers for Disease Control and Prevention indicates that the prevalence of obesity in children (including adolescents) and adults is 19.3% and 42.4%, respectively (www.cdc.gov, accessed on 5 April 2021). These figures cannot be explained by genetic risk factors alone and strongly suggest that environmental factors, including diet, are important contributors in the modern developed world [1, 2]. Obesity is an inflammatory disorder that involves both innate and adaptive immunity [3–10]. In addition to innate immune cells (including macrophages, natural killer cells and innate lymphoid cells), adaptive immune T and B lymphocytes are present in adipose tissues [11]. Moreover, treatment with anti-CD3 and anti-CD20 agents, which deplete T and B lymphocytes, respectively, ameliorates the metabolic syndrome, obesity and insulin resistance [3, 12].

IgM is the primary antibody produced by B cells in humans and mice on encountering antigens, particularly bacteria [13], and is evolutionarily conserved in all vertebrates, although the function varies between species. Obese individuals display elevated plasma IgM concentrations [14] but it is not clear how IgM affects obesity and metabolism. Activation-induced cytidine deaminase (AID) is a critical enzyme for immunoglobulin class-switch recombination to generate a diverse repertoire of antibodies. In the absence of AID, antibody repertoires are restricted to only IgM in both humans and mice [15], therefore AID-deficient mice provide a unique tool to investigate the role of IgM. Although studies of high-fat diet-induced obesity (HFDIO) in animal models have provided invaluable information, there are still knowledge gaps in our understanding of the role of the immune system in the chronic and systemic processes involved in obesity, insulin resistance and type 2 diabetes.

To investigate the immunometabolic role of IgM in obesity we studied the effects of HFDIO in *Aid*^−/−^ (also known as *Aicda*^−/−^) B6 mice, which have B cells but can make only IgM antibodies. We also assessed the proportion of IgM-bound gut microbiota in children with obesity, with or without type 2 diabetes. Our study provides a novel link between immunoglobulin (especially IgM), obesity and type 2 diabetes in mice and humans.

## Methods

### Mice

*Aid*^−/−^ C57BL/6 breeders were kindly provided by T. Honjo (Kyoto University, Japan) [16]. C57BL/6 breeders were purchased from the Jackson Laboratory (C57BL/6J [The Jackson Laboratory, USA; https://www.jax.org/strain/000664] RRID: IMSR_JAX:000664). The original *Aid*^−/−^ C57BL/6 breeders were bred with wild-type (WT) C57BL/6 breeders to obtain *Aid*^+/−^ C57BL/6 mice, and intercrossed, generating *Aid*^−/−^ C57BL/6. Both *Aid*^−/−^ C57BL/6 and WT C57BL/6 colonies have been maintained in the same room at Yale University for >10 years. Germ-free (GF) C57BL/6 breeders were kindly provided by R. Flavell (Yale University, USA) and expanded in the Yale gnotobiotic mouse facility. All the mice used in the study (except GF mice) were housed in specific pathogen-free conditions with autoclaved supplies, including food and bedding, and maintained in a 12 h dark–light cycle. The mice were fed with standard diet (Teklab Global, USA; no. 2018S, 6.2% fat) or high-fat diet (HFD) (Research Diet, USA; D12492, 60% fat). Mice from different litters were randomly assigned to experimental groups. All the animal procedures were approved by Yale Institutional Animal Care and Use Committee (IACUC).

### Human samples

Human stool samples were from children and adolescents who were obese with normal glucose tolerance (NGT), obese with impaired glucose tolerance (IGT) [17] or obese and newly diagnosed with type 2 diabetes (electronic supplementary material [ESM] Table 1, ESM Fig. 1). Fresh human stool samples were either snap-frozen or aliquoted in 50% glycerol and stored at −80°C. Donors were recruited from the Yale Pediatric Obesity Clinic and all the individuals from whom stool samples were collected were treatment-naive at the time of the sample collection. The study was approved by the Yale Human Investigation Committee. Written parental informed consent and written child assent were obtained from all the participants. Exclusion criteria included known hepatic diseases, alcohol consumption, smoking and medication use.

### HFDIO in mice

Male mice (6 weeks old) were fed with HFD (replenished twice weekly) for 4 months. Body weight was measured weekly. HFD food consumption was assessed daily by measuring the difference in weight between the food provided and that remaining after 24 h, including the food on the cage floor.

### IPGTT and ITT in mice

Mice were fasted overnight, with free access to drinking water, prior to the IPGTT. For the ITT, mice were fasted for 6 h, with drinking water available ad libitum. Baseline blood glucose was measured before i.p. injection of either glucose solution (1 g/kg glucose in sterile water) or insulin solution (0.75 U/kg; Humulin R; Lilly, USA). Blood glucose was measured at 15, 30, 60 and 90 min after glucose or insulin injection with a FreeStyle glucose meter (Abbott, USA); a glucose analyser (Analox Instruments, USA) was used if the reading was over the detectable range of the FreeStyle glucose meter.

### OGTT in humans

A standard OGTT (1.75 g/kg body weight, up to 75 g) was performed after participants had fasted overnight for 10–12 h. Blood samples were taken for determination of glucose and insulin over 180 min at the Yale Clinical Center Investigation in the Hospital Research Unit of the Yale New Haven Hospital.

### Faecal microbiota transfer in mice

Fresh faecal pellets were pooled from three or four mice on HFD for 16 weeks. Pellets were homogenised and centrifuged to remove large particles (1 *g* [~100 rev/min], 10 min). Bacterial colony-forming units (CFU) were measured by absorbance (OD600) with a pre-titrated control bacterium, *Escherichia coli*. GF C57BL/6 mice (4 weeks old) were colonised with faecal bacteria from HFDIO donor mice by oral gavage (2 × 10^8^ CFU/mouse). For faecal microbiota transfer (FMT) of human faecal material, frozen bacteria were quickly thawed in a 37°C water bath and washed twice in sterile PBS, before oral gavage. Four-week-old GF C57BL/6 mice were colonised with 2 × 10^8^ CFU of stool bacteria from individuals with NGT, IGT or type 2 diabetes, in 100 μl sterile PBS. Colonised mice were euthanised within 2 weeks post-gavage.

### Isolation of IgM-bound and unbound gut bacteria

The stool samples from eight study participants were washed in 1 ml filtered sterile PBS (226 *g*, 5 min), prior to resuspension of the bacterial pellet in 1% BSA/PBS. Bacteria were then incubated with 10 μl of rat serum (Sigma, USA) at room temperature for 15 min prior to incubation with an anti-human IgM antibody (MHM-88; BioLegend, USA) at 4°C for 30 min. Bacteria were washed twice with filtered sterile PBS prior to resuspension in PBS. IgM-bound (IgM^+^) and unbound bacteria from each sample were sorted using FACSAria (BD Biosciences, USA).

### Extraction of bacterial DNA, 16S rRNA sequencing and microbiota classification

Colonic contents or faecal pellets were collected from the mice on standard diet and HFD at the time of termination (16 weeks after commencement of HFD). DNA extraction and 16S rRNA sequencing was performed from both total gut microbiota and flow-cytometrically-sorted gut bacteria, as described previously [18] (see ESM Methods). The results were quality-filtered using Quantitative Insights Into Microbial Ecology (QIIME) software package (v1.8; [19]) with further quality and chimera-filtering in the UPARSE pipeline (v7.0; [20]). Operational taxonomic units (OTUs) were picked with 97% identity in the UPARSE pipeline. QIIME, using the Greengenes reference database for taxonomy assignment, was performed at various levels using representative sequences of each OTU. β diversity was calculated to compare differences between microbial community profiles, with data shown as principal component of analysis (PCoA). Predicted microbial functions were determined following phylogenetic investigation of communities by reconstruction of unobserved states (PICRUSt) analysis [21] from the 16S rRNA sequencing results. The 16S rRNA sequencing datasets supporting the current study have been deposited in the NCBI SRA public repository (accession no. SAMN18796639).

### Immune cell extraction from adipose tissue

Abdominal visceral fat tissue, harvested immediately after termination of the mice, in sterile conditions, was cut into ~1 mm pieces in a Petri dish and digested in collagenase I (1 mg/ml in RPMI-1640 medium) with shaking (250 rev/min) in an incubator at 37°C for 1 h. Digestion was stopped by adding 10 ml complete medium (RPMI-1640 medium with 5% FCS, 10 mmol/l 2-mercaptoethanol, penicillin–streptomycin–glutamine), followed by centrifugation at 1297 *g* for 10 min. After decanting the supernatant fraction, the cell pellet was resuspended in 2 ml ammonium-chloride-potassium (ACK) buffer, at room temperature for 5 min, to lyse residual erythrocytes. After adding 5 ml of complete medium, the cells were filtered through 100 μm nylon mesh followed by centrifugation (1297 *g*, 5 min). Immune cells in the cell pellet were resuspended and stained with monoclonal antibodies conjugated with various fluorochromes (see below).

### Immune cell isolation from spleen and lymphoid tissues

Spleen, mesenteric lymph nodes (MLN) and Peyer’s patches (PP) were dissected from mice and collected into individual tubes containing 3–5 ml PBS. All tissues were homogenised into a single-cell suspension using frosted microscope slides, prior to filtering through a nylon filter (150 μm) to remove debris. Erythrocytes in the loosened pellet of splenocytes were lysed by hypotonic ‘shock’ with 900 μl sterile water for a few seconds, immediately followed by 100 μl of 10× PBS, restoring isotonicity. The cells were filtered, washed and resuspended.

### Quantitative real-time PCR

RNA was extracted from mouse tissues (e.g. epididymal adipose tissue, liver, muscle and distal small intestine) using a Qiagen RNAeasy kit (Qiagen, Germany), prior to cDNA synthesis, following the manufacturer’s instructions (Bio-Rad). Quantitative real-time PCR (qPCR) was performed using a qPCR cycler (iQ5; Bio-Rad, USA), according to the manufacturer’s instructions. Primer sequences are listed in ESM Table 2. Relative gene expression was determined using the method by normalisation with *Gapdh* housekeeping gene.

### Antibody measurement from serum and intestinal flush

Serum and gut contents were collected from colonised GF C57BL/6 mice at 6 weeks old. The small intestine was flushed with 5 ml of sterile PBS, and the gut flush centrifuged (1297 *g*, 5 min, room temperature). The supernatant fraction was collected for antibody (IgA, IgM and IgG) detection using ELISA (Southern Biotech, USA) [22].

### Flow cytometry

Cells were stained with pre-determined dilutions of fluorochrome-conjugated monoclonal antibodies (BioLegend, unless specified) and analysed on a BD LSRII flow cytometer (see ESM Table 3).

### Antibodies and reagents

Purified mouse IgG (D609–0100) and IgA (010–001-341) were used for in vivo infusion (Rockland Immunochemicals, USA) (>95% purity, <2 U/mg endotoxin). All chemicals used in the study were from Sigma, unless specifically noted, with RPMI-1640 medium (Invitrogen, USA), heat-inactivated FBS and 100× stock solution of penicillin–streptomycin–glutamine (Gibco, USA) for cell culture.

### Gut permeability assay

Mice were fasted overnight before gavage with FITC–dextran (600 mg/kg; *M*_*r*_: 3000–5000) (Sigma). Baseline blood samples were collected prior to oral gavage with FITC–dextran in sterile PBS. Two hours post-gavage, food supply was restored to the mice. After a further 2 h, blood samples were collected and centrifuged (2300 *g*, 5 min, room temperature) to separate serum. Serum samples were diluted 1:1 in PBS in a 96-well plate and serum FITC–dextran concentration was measured by fluorescence spectrophotometer (Perkin Elmer, USA). Serum samples from non-FITC–dextran-gavaged mice were used as baseline. Standard curves were generated using known concentrations of FITC–dextran diluted in control serum. Concentrations were determined using linear regression.

### Infusion of IgG or IgA to *Aid*^−/−^ mice

Male *Aid*^−/−^ C57BL/6 (6 weeks old) were infused (i.v.) either with affinity-purified mouse polyclonal IgG or IgA (100 μg/mouse) during the first 2 weeks of HFD and during the last 2 weeks of 13 weeks’ HFD. IgG infusion was given twice a week and IgA infusion was given three times a week based on the t_½_ of mouse IgG and IgA, respectively [23, 24]. A group of mice was infused with PBS as controls.

### DEXA

Body composition of the HFDIO mice was measured by DEXA scan (Micro Photonics, Allentown, USA). Total body fat and the fat in the region of interest (ROI) were calculated with the software provided with the DEXA.

### Statistical analysis

Statistical analysis was performed using GraphPad Prism software V7 or V9 (GraphPad, USA). Differences between groups were analysed using a two-tailed Student’s *t* test or a two-way ANOVA. Differences between microbial species were determined following analysis using multiple *t* tests with false discovery rate correction; *p*<0.05 was considered significant.

### Randomisation, blinding and inclusion/exclusion criteria for murine studies

Mice were randomly assigned to experimental groups from multiple breeders. All experiments were conducted unblinded to the investigators with the exception of the experiments involving the study or use of the human samples (e.g. study of antibodies in human donor bacteria or bacterially gavaged GF mice). Mice were excluded from experiments if they were runts or developed signs of ill health.

## Results

### *Aid*^−/−^ mice are more susceptible to HFDIO and insulin resistance

**Fig. 1:**
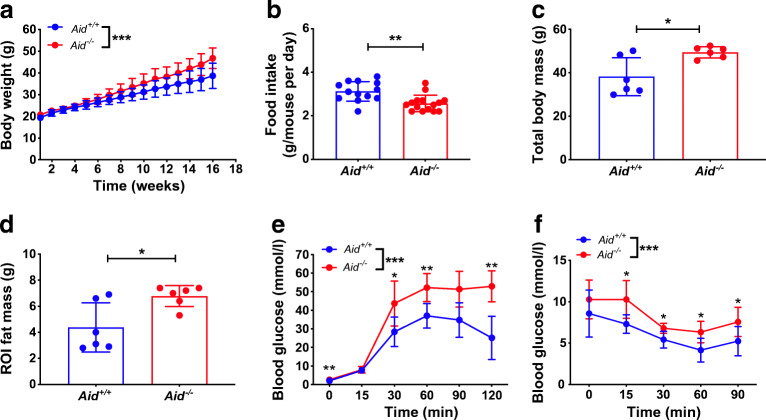
*Aid*^−/−^ mice are more susceptible to HFDIO and insulin resistance. Six-week-old male *Aid*^+/+^ or *Aid*^−/−^ mice were fed HFD for 16 weeks. (**a**) Longitudinal assessment of body weight over the 16 weeks of HFD. (**b**) Average food intake per mouse per day. (**c**, **d**) Body mass (**c**) and abdominal fat composition (**d**) assessed by DEXA scan after 16 weeks of HFD. (**e**, **f**) Glucose (**e**) and insulin (**f**) tolerance tests in HFD-fed mice. Data were pooled from two separate experiments, except for (**e**) and (**f**), which show one of three representative experiments. *n* = 13 *Aid*^+/+^ mice and *n* = 14 *Aid*^−/−^ mice (**a**); *n* = 6 mice/group (**e**, **f**). Data were assessed for significance using a two-way ANOVA (**a**, **e**–**f**) or Student’s *t* test (**b**–**d**). Data are presented as mean ± SD. *p<0.05, **p<0.01, ***p<0.001

*Aid*^−/−^ mice fed with standard food (NF, 6.2% fat) had normal insulin resistance and better glucose tolerance compared with *Aid*^+/+^ WT B6 mice, although their body weight was slightly higher (ESM Fig. 2). To assess the role of IgM in obesity, we fed male *Aid*^−/−^ B6 mice, which have B cells but can make only IgM antibodies, and *Aid*^+/+^ WT B6 mice with HFD (60% fat) for 16 weeks (from 6 weeks old). Male mice are known to be susceptible to diet-induced obesity, and the *Aid*^−/−^ mice were more susceptible to HFDIO, as they gained more body weight (Fig. 1a), although their food intake was lower than that of the *Aid*^+/+^ mice (Fig. 1b). We also scanned the mice by DEXA to determine the total body mass (fat and lean) and abdominal (ROI) fat composition. *Aid*^−/−^ mice had significantly higher body mass and abdominal fat (Fig. 1c, d). Next, we tested glucose and insulin tolerance in the *Aid*^−/−^ and WT mice following HFDIO, demonstrating that the *Aid*^−/−^ mice had IGT (Fig. 1e) and insulin resistance (Fig. 1f).

### More immune cell infiltrates and inflammatory cytokine-producing immune cells in visceral fat tissues and mucosal lymph nodes in *Aid*^−/−^ mice

**Fig. 2:**
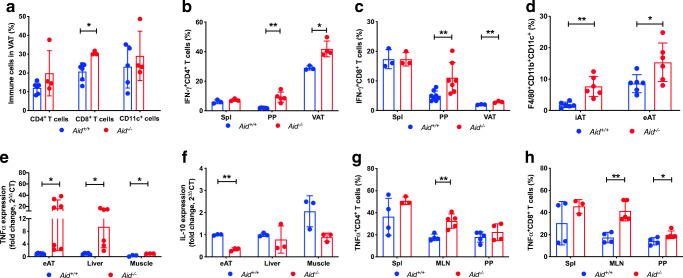
*Aid*^−/−^ mice have increased proinflammatory immune infiltration in VAT and mucosal lymph nodes. Six-week-old male *Aid*^+/+^ or *Aid*^−/−^ mice were fed HFD for 16 weeks prior to termination. (**a**) The proportion of immune-infiltrating CD4^+^ and CD8^+^ T cells and CD11c^+^ cells from VAT were determined by flow cytometry and gated from live single cells. (**b**, **c**) Cells from the spleen, PP and VAT were harvested and stimulated for 4 h in the presence of phorbol 12-myristate 13-acetate (PMA), ionomycin and GolgiPlug (BD Biosciences), prior to cell staining. The proportion of IFN-γ-producing CD4^+^ T cells (**b**) and IFN-γ-producing CD8^+^ T cells (**c**) gated as in **a**. (**d**) The proportion of macrophages in the inguinal and epididymal adipose tissue, gated from live, single CD11b^+^CD11c^+^F4/80^+^ cells. (**e**, **f**) Adipose tissue, liver and muscle were snap-frozen, prior to RNA extraction and qPCR for *Tnfα* (also known as *Tnf*) (**e**) and *Il-10* (also known as *Il10*) (**f**) gene expression analysis. Samples were averaged from triplicates with the relative gene expression determined using the method by normalisation with the housekeeping gene, *Gapdh*. (**g**, **h**) The proportion of TNF-α-secreting CD4^+^ (**g**) and CD8^+^ (**h**) T cells from the spleen, MLN and PP, investigated by flow cytometry and gated as in **a**. Data are pooled from two independent experiments, except for **e** and **f**, which show one representative of two experiments (*n* = 3–6). Data were assessed for significance using Student’s *t* test. Data are presented as mean ± SD. *p<0.05, **p<0.01. eAT, epididymal adipost tissue; iAT, inguinal adipose tissue; Spl, spleen

To investigate the immune cell infiltration in white fat tissue, we isolated immune cells from visceral adipose tissue (VAT) in the HFDIO *Aid*^−/−^ mice. The proportion of CD8^+^ T cells was significantly increased in the VAT of *Aid*^−/−^ mice compared with WT mice, while no significant differences were observed in CD4^+^ T cells or CD11c^+^ cells (Fig. 2a). The *Aid*^−/−^ mice had a significantly higher percentage of IFN-γ-producing CD4^+^ and CD8^+^ T cells in VAT and PP but no difference was observed in the spleen (Fig. 2b, c). Total B cells were also increased in the VAT of *Aid*^−/−^ mice but the difference did not reach statistical significance compared with WT mice; however, we found that CD1d^low^ B cells in VAT were significantly increased in *Aid*^−/−^ mice (ESM Fig. 3). Consistent with the other results, there was a highly significant increase in F4/80^+^CD11b^+^CD11c^+^ macrophages in both the inguinal and epididymal adipose tissue and all lymphoid tissues studied in *Aid*^−/−^ vs WT mice after HFD feeding (Fig. 2d and ESM Fig. 4). To determine whether the increased obesity found in HFDIO *Aid*^−/−^ mice was associated with inflammation in adipocytes, we examined *Tnfα* (also known as *Tnf*) mRNA in the adipose tissue, liver and muscle of *Aid*^−/−^ and WT mice. *Tnfα* mRNA was increased in *Aid*^−/−^ mice in all tissues tested, particularly in the epididymal adipose tissue and liver (Fig. 2e). In contrast, *Il-10* (also known as *Il10*) mRNA (an anti-inflammatory cytokine) was reduced in the epididymal adipose tissue and muscle tissues but not the liver of the *Aid*^−/−^ mice (Fig. 2f). We next examined the TNF-α-producing T cells in the lymphoid tissues, especially the gut-associated lymphoid tissues (MLN and PP). TNF-α-producing CD4^+^ and CD8^+^ T cells were significantly increased in HFDIO *Aid*^*−/−*^ mice in the MLN, with TNF-α-producing CD8^+^ T cells also increased in the PP; no differences were found in the spleen (Fig. 2g, h).

### Altered gut microbiota in *Aid*^−/−^ mice can transfer body weight gain and IGT to GF mice

**Fig. 3:**
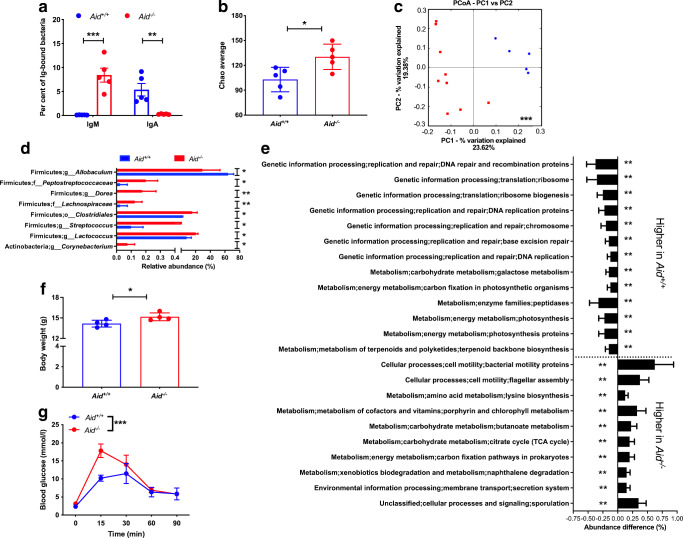
*Aid*^−/−^ mice have altered gut microbiota, which can transfer body weight gain and IGT to GF mice. Faecal pellets were collected at 12 weeks post-HFD from male *Aid*^+/+^ and *Aid*^−/−^ mice, prior to DNA extraction and investigation of microbial composition by 16S rRNA deep sequencing. (**a**) IgM- or IgA-bound gut bacteria were determined by flow cytometry after staining with fluorescence-conjugated anti-mouse IgM or IgA. (**b**) α diversity, as assessed by Chao average (to determine species richness). (**c**) β diversity, as assessed by PCoA. (**d**) Further microbial changes within the Firmicutes and Actinobacteria phyla. Changes were found at the order (o_Clostridiales), family (f_Peptostreptococcaceae and Lachnospiraceae) and genus (g_; all others) taxonomic levels. (**e**) Differences in predicted microbial functions following PICRUSt analysis. (**f**, **g**) Faecal microbiota from male *Aid*^+/+^ and *Aid*^−/−^ mice 14 weeks after HFD were isolated and transferred, by oral gavage, to GF C57BL/6 mice. Ten days later, recipient mice were investigated for changes in their body weight (**f**) and glucose tolerance (**g**). Data are from one of two independent experiments, except **b**–**e**, which were from one sequence run of the samples from two experiments. *n* = 4 or 5, except **c** where *n* = 5–9. Data were assessed for significance using Student’s *t* test (**a**, **b**, **f**), Analysis of similarities (ANOSIM) (**c**), multiple Student’s *t* test with false discovery rate correction (**d**, **e**) or two-way ANOVA (**g**). Data are presented as mean ± SEM (**a**) or mean ± SD (**b**–**g**). *p<0.05, **p<0.01, ***p<0.001

Polyreactive IgM antibodies may react to gut commensal bacteria, with most gut IgM deriving from plasma cells affiliated to memory IgM^+^ B cells, at least in humans [25]. We tested whether AID-deficiency altered the total bacterial community composition, by assessing IgM reactivity to gut bacteria in *Aid*^−/−^ mice. We collected faecal samples from *Aid*^−/−^ and WT mice after 12 weeks of HFD, by which time the *Aid*^−/−^ mice had gained significantly more body weight than WT mice (Fig. 1a), and investigated IgM-bound gut bacteria by flow cytometry. As predicted, *Aid*^−/−^ mice harboured a higher proportion of gut bacteria ‘coated’ with IgM compared with WT mice, whereas WT mice had more IgA-bound gut bacteria (Fig. 3a). To assess whether the increased obesity, impaired metabolic function and heightened inflammation in *Aid*^−/−^ mice were associated with changes in overall gut microbiota, we isolated bacterial DNA and performed 16S rRNA deep sequencing. *Aid*^−/−^ mice had a higher α diversity (Fig. 3b) and the composition of the gut microbiome (β diversity) in *Aid*^−/−^ mice was also very different from that in WT control mice (Fig. 3c). We also found that some bacterial species of the phylum Firmicutes were significantly different in *Aid*^−/−^ mice compared with those found in control WT mice (e.g. *Lactococcus*, *Streptococcus*, *Dorea*, all of which were increased in *Aid*^−/−^ mice) (Fig. 3d). Furthermore, by PICRUSt analysis [21] from the 16S rRNA sequencing data, we identified predicted microbial functions, which were either reduced or increased (Fig. 3e). Interestingly, these predicted microbial functions suggested enhanced tricarboxylic acid metabolism and bacterial motility/flagellar assembly in *Aid*^−/−^ mice compared with WT mice, whereas bacteria from WT mice had enhanced bacterial genetic information processing. To assess the biological function of the altered gut microbiota in *Aid*^−/−^ mice, we performed FMT experiments to determine whether gut microbiota were responsible for the metabolic dysfunction in *Aid*^−/−^ mice. We transferred faecal samples from *Aid*^−/−^ and WT mice (14 weeks post-HFD) to GF WT B6 mice (4 weeks old), maintaining standard diet before and after FMT, and examined body weight and IPGTT at 10 days post-FMT. We found that gut microbiota from donor *Aid*^−/−^ mice promoted body weight gain (Fig. 3f) and IGT (Fig. 3g) as early as 10 days post-FMT in otherwise healthy young recipient GF B6 mice on standard diet.

### Gut microbiota from obese individuals with type 2 diabetes induce metabolic dysfunction and a ‘leaky gut’

**Fig. 4:**
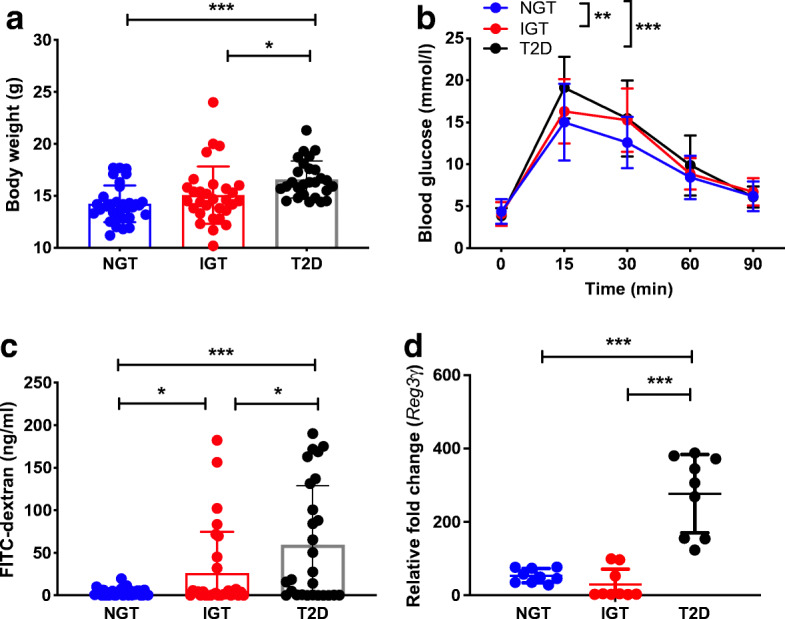
Gut microbiota from obese individuals with type 2 diabetes induce metabolic dysfunction and a more permeable gut. Faecal microbiota from human donors with NGT, IGT or type 2 diabetes (*n* = 5 donors/group) were transferred, by oral gavage, to GF C57BL/6 mice. (**a**, **b**) After 7 days, recipient mice (*n* = 32 NGT; *n* = 30 IGT; *n* = 27 type 2 diabetes) were investigated for changes in their body weight (**a**) and glucose tolerance (**b**). (**c**) At 9 days post-microbial gavage, recipient mice (*n* = 32 NGT; *n* = 30 IGT; *n* = 27 type 2 diabetes) were investigated for changes to their gut permeability by oral gavage of FITC–dextran. (**d**) At 12 days post-microbial gavage, small intestinal (ileal) tissue from recipient mice (*n* = 9 NGT; *n* = 9 IGT; *n* = 9 type 2 diabetes) was harvested prior to RNA extraction and qPCR for expression of antimicrobial peptide gene *Reg3γ*. Samples were averaged from triplicates, with the relative gene expression determined using the method by normalisation with the housekeeping gene, *Gapdh*. Data were pooled from three or more independent experiments and assessed for significance using Student’s *t* test (**a**, **c**, **d**) or two-way ANOVA (**b**). Data are presented as mean ± SD. *p<0.05, **p<0.01, ***p<0.001. T2D, type 2 diabetes

Our findings in mice prompted us to conduct FMT experiments using previously sequenced donor bacteria from treatment-naive individuals with NGT, IGT or type 2 diabetes to assess the metabolic function of the gut microbiota [17]. FMT was performed using the bacteria from each group of individuals, into the GF B6 mice using the same approach as for *Aid*^−/−^ mice described above. It is intriguing that the body weight of ex-GF B6 mice (i.e. GF mice after FMT) showed a stepwise increase, with the greatest weight gain observed in the mice receiving FMT from the obese individuals with type 2 diabetes, as early as 7 days post-colonisation (Fig. 4a), and mirrored by glucose intolerance (Fig. 4b). To probe the mechanism of metabolic dysfunction induced by the gut microbiota, we assessed gut permeability of the ex-GF mice. Interestingly, gut microbiota from obese individuals with type 2 diabetes induced the ‘leakiest’ gut in the ex-GF mice (i.e. having the highest concentration of circulating FITC–dextran, indicating increased gut permeability) (Fig. 4c), together with the highest levels of antimicrobial peptides, especially *Reg3γ*, in the intestine (Fig. 4d). In contrast, the GF mice colonised with microbiota from obese individuals with NGT presented with the lowest gut permeability.

### Gut microbiota from obese individuals with type 2 diabetes promote stronger humoral responses in GF mice

**Fig. 5:**
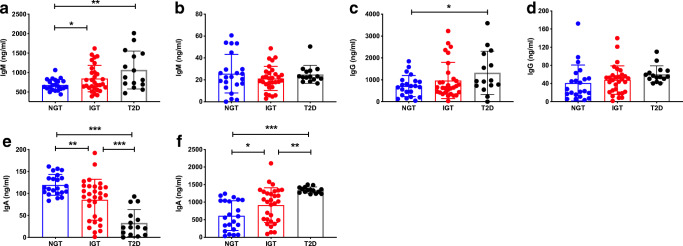
Gut microbiota from obese individuals with type 2 diabetes promote stronger humoral responses in GF mice. Faecal microbiota from obese human donors with NGT, IGT or type 2 diabetes (*n* = 5 donors/group) were isolated and transferred, by oral gavage, to GF C57BL/6 mice. After 12 days, serum and small intestinal contents were collected from recipient mice (*n* = 22 NGT; *n* = 30 IGT; *n* = 15 type 2 diabetes). IgM (**a**, **b**), IgG (**c**, **d**) and IgA (**e**, **f**) antibodies were measured in the serum (**a**, **c**, **e**) and small intestine (**b**, **d**, **f**) by ELISA. Data were pooled from three independent experiments. Data were assessed for significance using Student’s *t* test. Data are presented as mean ± SD. *p<0.05, **p<0.01, ***p<0.001. T2D, type 2 diabetes

To further investigate the functional effects of the transferred microbiota, we assessed the levels of immunoglobulins in serum and faecal samples of the ex-GF mice after FMT from humans. Ex-GF mice expressed the highest level of circulating IgM after receiving FMT from obese individuals with type 2 diabetes, when compared with the ex-GF mice receiving FMT from obese individuals with NGT (Fig. 5a), although the IgM level in the intestine was similar (Fig. 5b). A comparable pattern was found for IgG (Fig. 5c, d). Interestingly, unlike the IgM and IgG responses, serum IgA level was significantly reduced but free IgA was significantly increased in the small intestine in the ex-GF mice receiving FMT from obese individuals with type 2 diabetes (Fig. 5e, f). Taken together, our results suggest that gut microbiota from obese individuals with type 2 diabetes promote stronger IgM humoral responses.

### More gut bacteria in obese individuals with type 2 diabetes are ‘coated’ with IgM and the bacteria differ between individuals with type 2 diabetes vs NGT or IGT

**Fig. 6:**
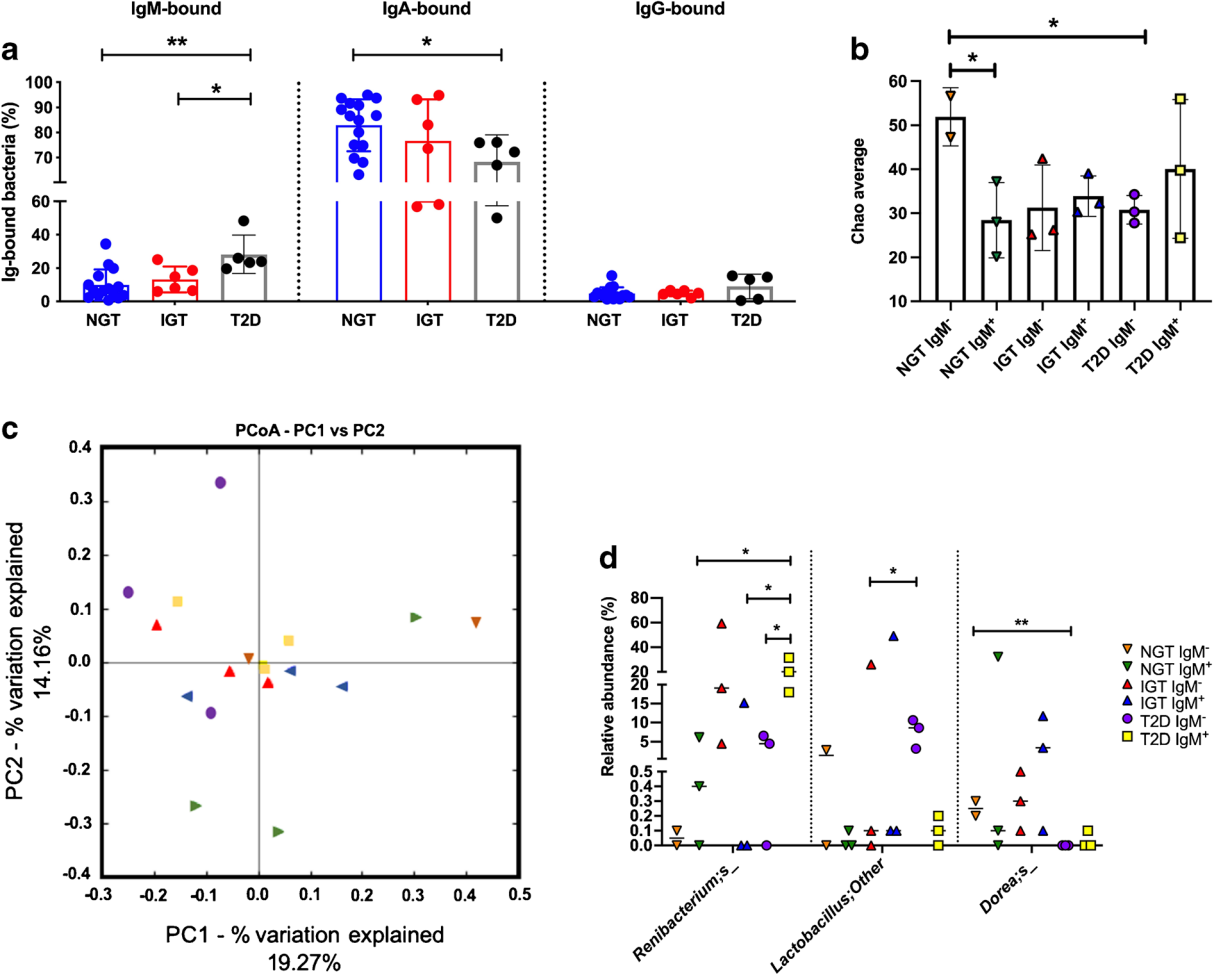
More gut bacteria in obese individuals with type 2 diabetes are IgM-coated and the bacteria differ between individuals with type 2 diabetes vs NGT or IGT. (**a**) The proportion of IgM-, IgA- and IgG-bound gut microbiota from obese individuals with NGT (*n* = 15), IGT (*n* = 6) or type 2 diabetes (*n* = 5) was measured by flow cytometry. (**b**–**d**) IgM-bound and unbound bacterial sequencing results: α diversity, as assessed by Chao average (to determine species richness) (**b**); β diversity, as assessed by PCoA (**c**); and relative abundance of bacterial species, as determined by 16S rRNA sequencing analysis (**d**). All faecal samples were collected from treatment-naive individuals. Orange triangles, red triangles, purple circles represent IgM^−^ bacteria from obese donors with NGT, IGT and type 2 diabetes, respectively; green triangles, blue triangles and yellow squares represent IgM^+^ bacteria from obese donors with NGT, IGT and type 2 diabetes, respectively. Data were assessed for significance using Student’s *t* test (**a**, **b**), Analysis of similarities (ANOSIM) (**c**) and multiple *t* tests (**d**). Data are presented as mean ± SD. *p<0.05, **p<0.01. T2D, type 2 diabetes

To determine whether IgM-bound gut bacteria are indeed associated with obesity, we analysed IgM-bound gut bacteria from obese paediatric patients with NGT, IGT or type 2 diabetes. Interestingly, we found that the proportion of IgM-bound gut microbiota increased stepwise, the highest level being in obese individuals with type 2 diabetes (Fig. 6a). We also observed a reduced proportion of IgA-bound bacteria in individuals with type 2 diabetes compared with those who had NGT. No differences in IgG-bound bacteria were noted when comparing the groups of participants. However, only IgM-bound bacteria were present in significantly higher proportions in the obese individuals with type 2 diabetes compared with those having NGT and IGT. To further dissect the composition of IgM-bound (IgM^+^) and non-IgM-bound (IgM^−^) gut bacteria, we randomly selected eight study participants (two control participants with NGT, three obese participants with IGT and three with type 2 diabetes) and flow-cytometrically sorted IgM^+^ and IgM^−^ gut bacteria from their stool samples, followed by sequencing. We found that IgM^−^ gut bacteria (including IgA^+^, IgG^+^ and non-Ig-bound bacteria) from participants with NGT had higher α diversity, compared with participants with either IGT or type 2 diabetes (Fig. 6b). In contrast to IgM^−^ bacteria, there was no difference in the α-diversity of IgM^+^ bacteria among the three groups. Assessment of β diversity by PCoA revealed no significant differences among the groups (Fig. 6c). However, further analysis at the species level revealed three bacterial species for which proportions differed between the groups (Fig. 6d). *Renibacterium* species were significantly increased in IgM^+^ bacteria in people with type 2 diabetes compared with the IgM^+^ bacteria in NGT and IGT groups, while two bacterial species (a *Dorea* species and a *Lactobacillus* species) differed in abundance in the IgM^−^ bacteria between individuals with NGT and type 2 diabetes (*Dorea*) and IGT and type 2 diabetes (*Lactobacillus*). Thus, it is possible that IgM-targeted bacteria may be important in contributing to the development of obesity or type 2 diabetes.

### IgG infusion into Aid^−/−^ mice ameliorates the exacerbated HFDIO and promotes Treg cells in adipose tissue

**Fig. 7:**
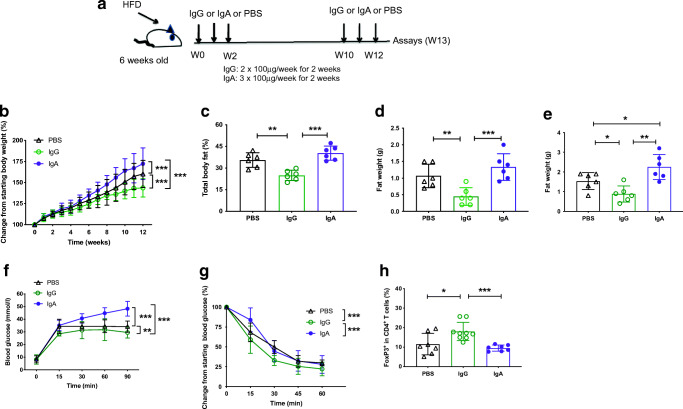
IgG infusion in *Aid*^−/−^ mice ameliorates the exacerbated HFDIO and promotes Treg cells in adipose tissue. Six-week-old male *Aid*^+/+^ and *Aid*^−/−^ mice were fed with HFD for 13 weeks and received purified polyclonal IgG, IgA or PBS by i.v. injection during the first two and last two weeks of the diet. (**a**) Depiction of the study design. (**b**) Proportional body weight change post-injection compared with starting body weight. (**c**–**e**) Proportion of total body fat assessed by Micro-CT (**c**) and weight of inguinal adipose tissue (**d**) and epididymal adipose tissue (**e**). (**f**, **g**) In vivo glucose (**f**) and insulin (**g**) tolerance responses from IgG-, IgA- or PBS-infused mice. Proportion of infiltrating Treg cells in the visceral fat, identified by flow cytometry, gated from live, single CD4^+^ T cells prior to gating on FoxP3^+^ cells (**h**). Data shown are pooled from two independent experiments. *n* = 6–8 per group. Data were assessed for significance using Student’s *t* test (**c**–**e**, **h**) or two-way ANOVA (**b**, **f**, **g**). Data are presented as mean ± SD. *p<0.05, **p<0.01, ***p<0.001. W, week

To further confirm the role of IgM in the exacerbated HFDIO, we infused mouse IgG or IgA into *Aid*^−/−^ mice when HFD feeding commenced (Fig. 7a). Interestingly, IgG ameliorated the aggravated obesity in *Aid*^−/−^ mice, whereas IgA appeared to further exacerbate HFDIO (Fig. 7b). The body fat composition and visceral fat were in line with the total body weight (Fig. 7c–e). The ameliorated obesity in the mice receiving IgG infusion was accompanied by improved glucose tolerance and insulin sensitivity, whereas enhanced HFDIO following IgA infusion was associated with greater impairment of glucose intolerance and insulin sensitivity (Fig. 7f, g). To investigate the mechanism by which IgG infusion ameliorated the exacerbated HFDIO, we examined immune cells in different tissues. Interestingly, we found a significant increase in CD4^+^Foxp3^+^ Treg cells in the adipose tissue in the IgG- but not IgA-infused *Aid*^−/−^ mice (Fig. 7h). However, Treg cells from the lymphoid tissues, including spleen, MLN and PP, were comparable, with or without IgG infusion (ESM Fig. 5). No differences in B cells or F4/80^+^CD11b^+^CD11c^+^ macrophages were found in any of the tissues examined in the *Aid*^−/−^ mice, with or without IgG infusion (data not shown).

## Discussion

In this study, we identified a potential novel role for IgM in the immunopathogenesis of obesity, mediated through altered gut microbiota, in both mice and humans and made three significant findings. First, *Aid*^−/−^ B6 mice, which can make only IgM and hence have elevated IgM levels, displayed exacerbated HFDIO and enhanced inflammation. Importantly, we also found altered gut microbiota coated with IgM in HFDIO. Second, the metabolic and immunological phenotype seen in obese *Aid*^−/−^ B6 mice could be transferred by the altered gut microbiota to GF WT B6 mice. Third, and perhaps most clinically relevant, youth with obesity and IGT or type 2 diabetes had increased faecal IgM and gut microbiota coated with IgM. Similar to the finding in *Aid*^−/−^ B6 mice, gut microbiota from children with obesity and IGT or type 2 diabetes could transfer the clinical metabolic features to GF WT B6 mice. Thus, we have identified a novel role for IgM in promoting obesity and inflammatory responses, mediated by IgM-associated gut microbiota.

Previous studies reporting that B cells play a role in obesity used B cell-deficient mice, either by genetic targeting or by drug depletion, could not distinguish the cellular from soluble antibody-production roles of B cells [12, 26]. Moreover, the humoral role of B cells, in particular the production of IgM, has not been studied in childhood obesity and childhood metabolic dysfunction or type 2 diabetes. IgM is a large molecule, with five monomers linked by a joining chain giving a pentameric structure that can bind more antigens and effectively activate complement [27, 28]. Here we used AID-deficient mice to provide a unique tool to investigate the role of IgM, as the absence of AID leads to antibody repertoires solely restricted to IgM in humans and mice [15]. This linked with our finding of increased faecal IgM and gut microbiota coated with IgM in youth with obesity and IGT or type 2 diabetes. Interestingly, in another context, it has been reported that fat-associated lymphoid clusters control local IgM secretion during lung inflammation [29].

IgA in obesity has been studied using an IgA-deficient (*Igha*^−/−^) mouse [30]. However, selective IgA deficiency in humans increases IgM [31]. Our finding in AID-deficient mice complements the *IgA*^−/−^ study by Luck et al [30], as AID-deficient mice are also deficient in IgA. Interestingly, in contrast to the report by Luck et al [30], when we infused IgA into AID-deficient mice, HFDIO worsened in the IgA-reconstituted *Aid*^−/−^ mice. The discrepancy may be due to the fact that *IgA*^−/−^ mice are deficient in both non-secreted IgA (mostly monomer in circulation) and secreted IgA (mostly dimer in mucosal compartments such as gut), whereas we reconstituted the mice with only monomeric IgA, which may have influenced the results. However, reconstitution with IgG led to amelioration of obesity and obesity-associated inflammation in AID-deficient mice, likely mediated by restoration of immune homeostasis by adipose tissue-resident Treg cells.

Increasing evidence suggests that gut microbiota play an important role in obesity and type 2 diabetes in humans and in mouse models [1, 17, 32–36]. The composition and metabolism of the gut microbiota is in large part driven by diet. Changes in gut microbiota induce a proinflammatory environment that occurs, in part, by increasing bacterial products, especially lipopolysaccharide, in blood and adipose tissue, and this can also promote insulin resistance [37]. Gut bacteria are immunogenic and stimulate B cells. The production of mucosal antibodies to the gut bacteria by B cells plays an important role in local, as well as systemic, immune tolerance or intolerance. IgM is the first antibody to be secreted during an immune response, prior to class switching (e.g. IgG, IgA), and upon binding to bacteria can be recognised by Fc receptors on cells (particularly innate immune cells like macrophages) resulting in destruction of the bacteria. In addition, the binding of antibody to bacteria can also limit bacterial growth and functions and thus antibody–bacteria binding can modulate gut microbiota composition, which in inflammatory bowel disease is associated with both disease development and activity [38, 39]. Thus, secreted antibodies modulate both microbes and the innate and adaptive immune response. Given that AID-deficient mice are unable to class-switch, this limits the ability of B cell antibodies to sufficiently modulate the gut microbiota composition, leading to weight gain and obesity. Similarly, in our study in humans, an increase in IgM-bound bacteria was associated with the development of type 2 diabetes. While HFD alters the gut microbiota composition to a pro-obesogenic profile, IgM, as the first antibody secreted, may try to restore intestinal homeostasis through targeting the microbiota; however, IgM alone is unable to modulate the microbiota composition sufficiently to an anti-obesogenic profile, which IgG was able to correct. IgM-restriction alters gut microbiota in *Aid*^−/−^ mice, promoting inflammation under the HFD-induced metabolic stress and in turn attracting inflammatory CD4^+^ and CD8^+^ T cells to the adipose tissue and gut mucosal-associated lymphoid tissue. Those T cells have effector memory phenotype and produce various inflammatory cytokines. The increase in the number of IFN-γ-producing T cells in AID-deficient mice could occur in relation to the specific stimulation by the microbiota and the inflammatory environment induced within the gut, and a response to the metabolic changes/obesity. Furthermore, macrophages are very important cells in obesity, also giving rise to inflammation, and we have observed systemic changes in macrophages. These may induce the inflammatory response of the T cells as well as provide the environment to increase the IgM-restricted B cells.

Magri et al recently reported that human intestine contains many IgM^+^ B cells that produce secretory IgM and intestinal IgM, which react to a variety of microbiota [25]. Given that IgM has a pentameric structure, it has more antigen binding sites than IgG and IgA, and thus IgM can bind more bacterial antigens and possibly different bacteria. Hence, IgM-bound gut microbiota are likely to induce stronger systemic immune responses in the host, which in turn could affect the energy metabolism of the host. Our study revealed that young individuals with obesity, especially those with metabolic dysfunction or clinical type 2 diabetes, have increased IgM-bound gut microbiota, the composition of which was different from non-IgM-bound gut microbiota. We observed that these microbiota not only induced a strong systemic IgM immune response in GF mice but also transferred aspects of the clinical metabolic phenotype. Our results provide further support for the role of B cells in obesity and type 2 diabetes [12, 26] and suggest a novel role for IgM and IgM-reactive gut microbiota in the immunopathogenesis of childhood obesity and type 2 diabetes. It is reasonable to speculate that similar metabolic inflammation and immune responses may occur in adult obesity and obesity-associated type 2 diabetes. The knowledge gained from our study may aid the development of novel therapeutics such as immunoglobulin therapy for treating obesity and type 2 diabetes.

## Supplementary information

ESM (PDF 411 kb)

## Data Availability

The 16 s rRNA sequencing datasets supporting the current study have been deposited in the NCBI SRA public repository (accession no. SAMN18796639).

## Abbreviations

AID: Activation-induced cytidine deaminase
CFU: Colony-forming units
FMT: Faecal microbiota transfer
GF: Germ-free
HFD: High-fat diet
HFDIO: High-fat-diet-induced obesity
IGT: Impaired glucose tolerance
MLN: Mesenteric lymph nodes
NGT: Normal glucose tolerance
OTU: Operational taxonomic unit
PCoA: Principal component of analysis
PICRUSt: Phylogenetic investigation of communities by reconstruction of unobserved states
PP: Peyer’s patches
QIIME: Quantitative insights into Microbial Ecology
qPCR: Quantitative real-time PCR
ROI: Region of interest
VAT: Visceral adipose tissue
WT: Wild-type
