# Supplementary material for: IgM-associated gut bacteria in obesity and type 2 diabetes in C57BL/6 mice and humans
Source: Diabetologia. 2022 May 19;65(8):1398–411. doi: 10.1007/s00125-022-05711-8 (PMC9283171; doi:10.1007/s00125-022-05711-8)
Supplement: Supplementary file 1 — (PDF 411 kb) [file 125_2022_5711_MOESM1_ESM.pdf]

Electronic Supplementary Materials:

ESM Methods
ESM Table 1 - Obese human youth microbial stool donor information.
ESM Table 2 - qPCR primer sequence details
ESM Table 3 – Flow cytometry monoclonal antibody details
ESM Fig. 1 - Glucose tolerance test results from the obese human youth microbial stool donors
ESM Fig. 2 – Body weight, glucose tolerance and insulin tolerance results from standard food-fed *Aid*$^{-/-}$ mice and WT mice
ESM Fig. 3 – B cells in the VAT
ESM Fig. 4 – Macrophages are increased in the lymphoid tissue of AID-deficient mice
ESM Fig. 5 – IgG infusion of *Aid*$^{-/-}$ mice does not alter Treg cells proportion from lymphoid tissues

ESM Methods

Extraction of gut bacterial DNA

For investigation of the total microbial community, colonic contents or fecal pellets were collected from the mice on both standard and HFD at the time of termination (16 weeks after commencement of HFD). To extract the bacterial DNA, fecal samples (~15-25mg/mouse) were re-suspended in 300µl Tris-EDTA and incubated for one hour at 37°C in the presence of 7.5µl Sodium dodecyl sulfate (0.5%) and 3µl Proteinase K (20mg/ml). One volume of phenol:chloroform:isoamyl alcohol (25:24:1), 200µl of 20% SDS and 0.3g of zirconium/silica beads (0.1mm, Biospec Inc, USA) were added and samples were mixed with a Mini-bead-beater for 2mins. The sample was then mixed with 820µl of phenol:chloroform:isoamyl alcohol (25:24:1), centrifuged and the aqueous layer collected into a new tube. The bacterial DNA was precipitated with 0.6 volumes of isopropanol, washed with 70% ethanol, air-dried and re-suspended in 100µl of sterile water.

16S rRNA sequencing

The V4 region of the bacterial 16S ribosomal gene was amplified from each DNA sample with barcoded, broadly conserved bacterial primers (forward, 5'- CATGCTGCCTCCCGTAGGAGT-3'; reverse, 5'-TCAGAGTTTGATCCTGGCTCAG-3'). PCR products were purified (QIAGEN gel extraction kit) and quantified (Nanodrop spectrophotometer), and equimolar amounts of each sample were pooled and pyrosequenced on an Ion Torrent Personal Genome Machine (PGM) sequencing system (Life Technologies, USA).

ID	Medications	Group	Age (years)	Sex	Race	Height (cm)	Weight (kg)	BMI	Body fat (%)	HbA_{1C} (mmol/mol)	HbA_{1C} (%)	Cholesterol (mmol/l)	HDL Cholesterol (mmol/l)	LDL Cholesterol (mmol/l)	Triglycerol (mmol/l)	ALT (U/l)	AST (U/l)
10	None	NGT	13.6	M	Hispanic	166	104.6	37.96	44.8	32.2	5.1	9.1	1.5	12.5	5.1	88	50
11	*None*	*NGT*	*12.5*	*M*	*Hispanic*	*163*	*81.4*	*30.64*	*47.4*	*41.0*	*5.9*	*5.9*	*1.8*	*3.1*	*4.9*	*24*	*19*
13	*None*	*NGT*	*13.2*	*F*	*African American*	*169*	*98.2*	*34.38*	*44.9*	*35.5*	*5.4*	*10.1*	*2.9*	*6.7*	*2.8*	*14*	*19*
28	Singlulair	NGT	9.9	M	Caucasian	148	61.4	28.03	48.4	39.9	5.8	9.7	2.9	6.3	2.2	11	21
29	Flovent inhaler; Salbutamol (Albuterol); Zyrtec	NGT	9.7	F	Caucasian	154	104.2	43.94	53.7	36.6	5.5	7.7	1.9	4.5	6.2	22	21
31	None	NGT	8	M	Caucasian	154	85	35.84	50.2	38.8	5.7	7.3	2.7	3.7	4.3	20	23
36	Lamictal, Topamax, Carbitrol	NGT	15.1	F	Caucasian	166	104.6	37.96	50.1	34.4	5.3	8.9	2.7	5.3	4.7	11	12
52	Pulacort; Singulair	NGT	7	F	Hispanic	143	73.6	35.99	46.7	34.4	5.3	7.8	1.7	4.9	6.0	34	29
57	None	NGT	12.4	M	Caucasian	166	108.2	39.27	58.4	34.4	5.3	10.2	2.4	6.4	6.6	33	22
58	None	NGT	15.2	M	Caucasian	182	109.2	32.97	33	35.5	5.4	6.9	2.6	3.4	4.4	55	52
60	None	NGT	13.1	M	African American	174	94.4	31.18	45.1	27.9	4.7	8.9	2.5	4.6	9.1	9	14
66	Salbutamol (Albuterol); Flovent; Metformin; Singulair; Nasonex	NGT	11.7	F	Hispanic	177	144	45.96	n/a	34.4	5.3	7.0	2.2	4.1	3.6	12	15

69	Salbutamol (Albuterol); Singulair; Symbacort	NGT	13	M	Hispanic	161	86.1	33.22	36.8	35.5	5.4	7.7	2.9	3.6	5.9	20	25
70	Salbutamol (Albuterol); Resta pump; Claritin	NGT	13.9	M	African American	173	115	38.42	48.8	36.6	5.5	7.8	2.6	4.8	2.3	19	21
78	Hydrocortisone	NGT	11.4	F	Hispanic	157	115.4	46.82	57.9	33.3	5.2	6.7	2.6	3.1	4.7	10	6
7	*None*	*IGT*	*14.7*	*F*	*African American*	*171*	*115.6*	*39.53*	*52*	*42.1*	*6*	*8.1*	*2.5*	*4.2*	*6.6*	*66*	*34*
8	None	IGT	13.6	F	Hispanic	163	90.6	34.1	45.7	43.2	6.1	9.1	2.3	5.1	8.4	40	32
32	None	IGT	7.2	F	Caucasian	122	29.6	19.89	29.6	35.5	5.4	5.5	2.8	1.8	4.4	39	36
38	*None*	*IGT*	*13.5*	*M*	*Hispanic*	*173*	*98.8*	*33.01*	*45.8*	*41.0*	*5.9*	*9.8*	*3.7*	*5.2*	*4.7*	*23*	*24*
46	Flovent; Salbutamol (Albuterol)	IGT	16.3	F	African American	160	93	36.328125	50.1	37.7	5.6	10.1	3.0	6.4	3.3	10	16
63	Cipro; Bactrim	IGT	15.6	M	Hispanic	173	102.8	34.34795683	49.4	38.8	5.7	12.3	2.7	7.5	10.9	37	25
72	*None*	*IGT*	*12.1*	*M*	*Hispanic*	*163*	*81.1*	*30.52*	*46*	*6.8*	*5.9*	*10.7*	*2.7*	*6.6*	*7.2*	*29*	*27*
65	None	T2D	20.1	M	Hispanic	179	123.2	38.45	29.1	38.8	5.7	10.2	1.4	5.5	27.5	57	27
82	*None*	*T2D*	*10.5*	*F*	*African American*	*153*	*122*	*51.28*	*54*	*45.4*	*6.3*	*8.7*	*2.5*	*4.4*	*11.1*	*42*	*28*
104	*None*	*T2D*	*11*	*M*	*African American*	*171*	*107.5*	*36.76*	*56*	*48.6*	*6.6*	*6.9*	*2.0*	*3.6*	*3.3*	*20*	*22*
116	None	T2D	8	F	Hispanic	136	72.1	38.98	51.5	54.1	7.1	8.8	2.1	5.8	4.6	23	23

120	*None*	*T2D*	*18*	*M*	*African American*	*163*	*119*	*45.38*	*48.8*	*61.7*	*7.8*	*8.2*	*2.4*	*4.8*	*4.8*	*20*	*25*

ESM Table 1. Obese human youth microbial stool donor information. Donors were recruited from the Yale Pediatric Obesity Clinic prior to receiving any medication. Individuals were assessed for glucose (see Fig. S1), lipid metabolism and liver function (ALT/AST). Stool donors were grouped into one of three groups (n=5/group) depending on their glucose tolerance results. Individuals in yellow indicate those microbial donors used for FMT experiments, while those in italics indicate those from which IgM+ and IgM- bacteria were sorted from for analysis. Abbreviations include: Normal glucose tolerant (NGT), Impaired glucose tolerant (IGT), Type 2 diabetes (T2D), Male (M), Female (F), Body mass index (BMI), Hemoglobin (Hb), High-density lipoprotein cholesterol (HDL-cholesterol), low-density lipoprotein cholesterol (LDL-cholesterol), Alanine aminotransferase (ALT) and Aspartate aminotransferase (AST).

Gene Name	Forward Primer Sequence (5'-3')	Reverse Primer Sequence (5'-3')
Tumor necrosis factor α (Tnfα)	CAAATGGCCTCCCTCTCAT	TGGGCTACAGGCTTGTCACT
Interleukin-10 (Il-10)	TGAATTCCCTGGGTGAGAAG	TCACTCTTCACCTGCTCCACT
Glyceraldehyde 3-phosphate dehydrogenase (Gapdh)	TGACATCAAGAAGGTGGTGAAG	TGCTGTAGCCGTATTCATTGTC
Reg3γ	TTCCTGTCCTCCATGATCAAAA	CATCCACCTCTGTTGGGTTCA

ESM Table 2. qPCR primer sequence details. A list of the six qPCR primer sequences used in the murine study.

Antibody Target	Clone	Supplier	Catalogue No.	RRID
CD1d	K253	BioLegend	140805	AB_10643277
CD4	GK1.5	BioLegend	100428	AB_493647
CD8α	53-6.7	BioLegend	100722	AB_312761
CD11b	M1/70	BioLegend	101224	AB_755986
CD11c	N418	BioLegend	117318	AB_493568
CD19	6D5	BioLegend	115530	AB_830707
CD45	30-F11	BioLegend	103132	AB_893340
FoxP3	FJK-16s	eBioscience	12-5773-82	AB_465936
IFNγ	XMG1.2	BioLegend	505810	AB_315404
TCRβ	H57-597	BioLegend	109220	AB_893624
TNFα	MP6-XT22	BioLegend	506313	AB_493328
Human IgM	HMH-88	BioLegend	314508	AB_493005
Viability Dye	not applicable	BioLegend	423102	

ESM Table 3. Monoclonal antibodies used for flow cytometry. A list of the mAbs used in the murine studies.

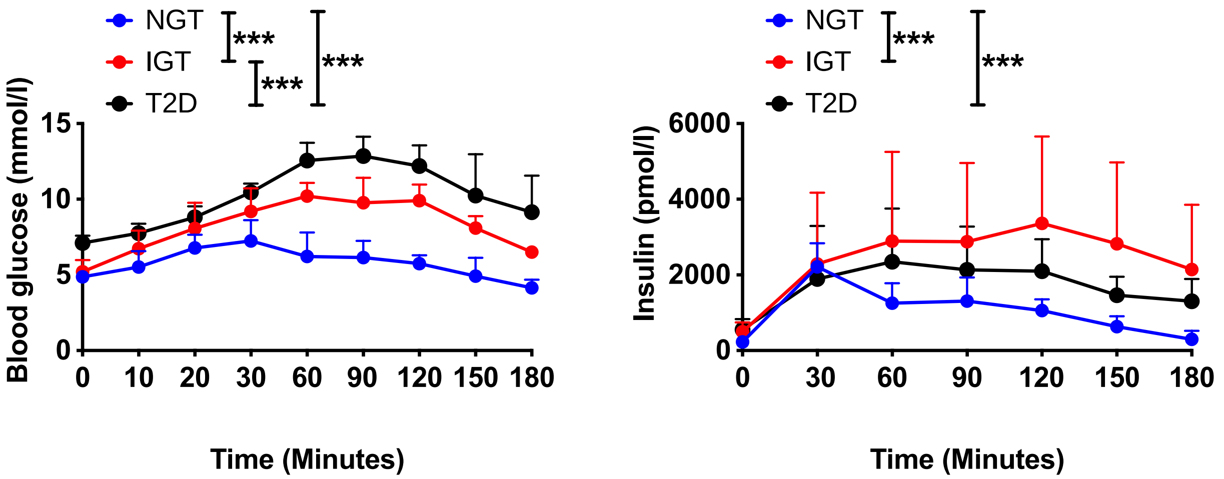

ESM Fig. 1 – Glucose tolerance test results from the obese human youth microbial stool donors

Individuals were fasted for 12 hours prior to oral administration of glucose and sequential testing of blood glucose (a) and insulin levels (b) over 180mins. Data were assessed for significance using a Two-way ANOVA. Data are presented as mean±SD. *** $p<0.001$.

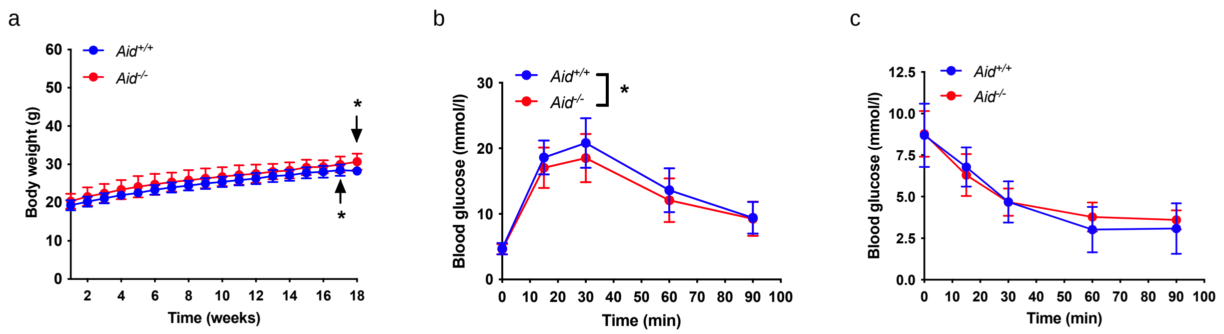

ESM Fig. 2 – Body weight, glucose tolerance and insulin tolerance results from standard food-fed *Aid*$^{-/-}$ mice and WT mice

a. Longitudinal assessment of body weight (g). Glucose (b) and insulin (c) tolerance tests in standard food-fed mice. Data were pooled from 2 separate experiments (n=13-14). Data were assessed for significance using a Student's t-test (a) or a Two-way ANOVA (b-c). Data are presented as mean±SD. * $p<0.05$.

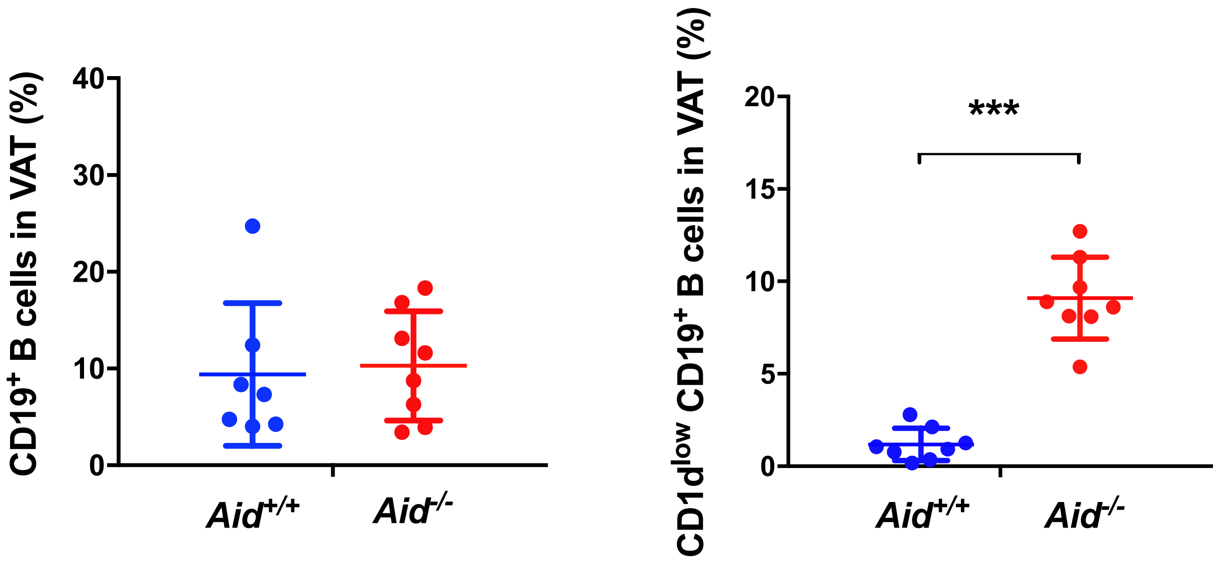

ESM Fig. 3 – B cells in VAT

a. The proportion of immune-infiltrating CD19^{+} B cells in the VAT from male *Aid*$^{+/+}$ or *Aid*$^{-/-}$ mice fed a high-fat diet (HFD) for 16 weeks. b. The proportion of CD1d^{low} CD19^{+} B cells in VAT. Data were pooled from 2 separate experiments and were assessed for significance using a Student's t-test. Data are presented as mean±SD. *** $p<0.001$.

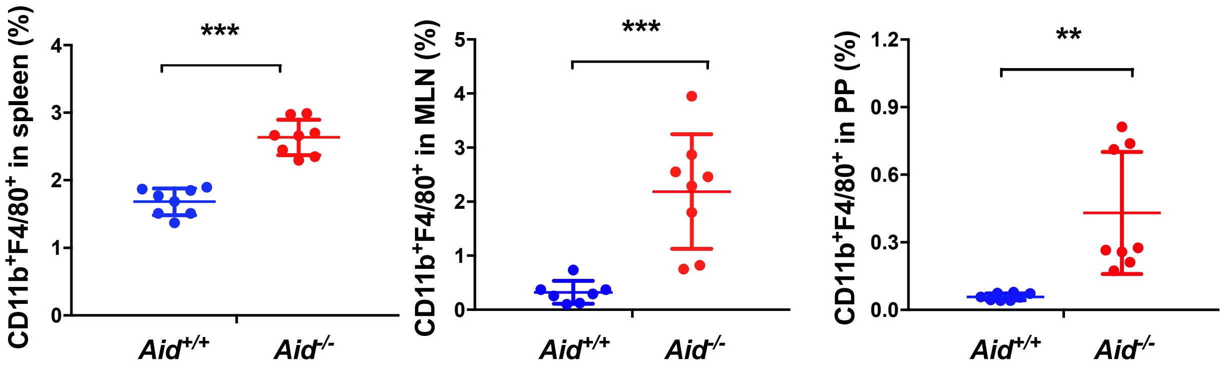

ESM Fig. 4 – Macrophages are increased in lymphoid tissue of AID-deficient mice

The proportion of $CD11b^+F4/80^+$ cells in the VAT from male *Aid*$^{+/+}$ or *Aid*$^{-/-}$ mice fed a high-fat diet (HFD) for 16 weeks. Data were pooled from 2 separate experiments and were assessed for significance using a Student's t-test. Data are presented as mean±SD. ** $p<0.01$, *** $p<0.001$.

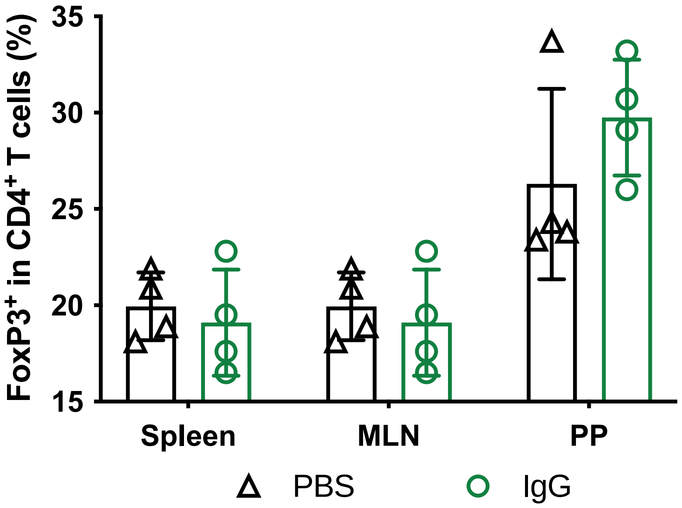

ESM Figure 5 – IgG infusion of *Aid*$^{-/-}$ mice does not alter Treg cells proportion from lymphoid tissues

Proportion of infiltrating Treg cells in the spleen, mesenteric lymph node or Peyer's patches from HFD fed male *Aid*$^{-/-}$ mice i.v. injected with either purified polyclonal IgG or PBS. Treg cells were identified by flow cytometry, gated from live, single CD4^{+} T cells prior to gating on FoxP3^{+} cells. Data shown are from 1 of 2 experiments (n=4). Data were assessed for significance using a Student's t-test. Data are presented as mean±SD.
